# Supplementary material for: Analytical validity of nanopore sequencing for rapid SARS-CoV-2 genome analysis
Source: Nat Commun. 2020 Dec 9;11:6272. doi: 10.1038/s41467-020-20075-6 (PMC7726558; doi:10.1038/s41467-020-20075-6)
Supplement: Supplementary file 12 — Reporting Summary [file 41467_2020_20075_MOESM12_ESM.pdf]

nature research

Corresponding author(s): Dr Ira Deveson (NCOMMS-20-30414)

Last updated by author(s): 10 November 2020

Reporting Summary

Nature Research wishes to improve the reproducibility of the work that we publish. This form provides structure for consistency and transparency in reporting. For further information on Nature Research policies, see our Editorial Policies and the Editorial Policy Checklist.

Statistics

For all statistical analyses, confirm that the following items are present in the figure legend, table legend, main text, or Methods section.

n/a	Confirmed	
☐	☒	The exact sample size (n) for each experimental group/condition, given as a discrete number and unit of measurement
☐	☒	A statement on whether measurements were taken from distinct samples or whether the same sample was measured repeatedly
☒	☐	The statistical test(s) used AND whether they are one- or two-sided<br>*Only common tests should be described solely by name; describe more complex techniques in the Methods section.*
☒	☐	A description of all covariates tested
☒	☐	A description of any assumptions or corrections, such as tests of normality and adjustment for multiple comparisons
☐	☒	A full description of the statistical parameters including central tendency (e.g. means) or other basic estimates (e.g. regression coefficient) AND variation (e.g. standard deviation) or associated estimates of uncertainty (e.g. confidence intervals)
☒	☐	For null hypothesis testing, the test statistic (e.g. F, t, r) with confidence intervals, effect sizes, degrees of freedom and P value noted<br>*Give P values as exact values whenever suitable.*
☒	☐	For Bayesian analysis, information on the choice of priors and Markov chain Monte Carlo settings
☒	☐	For hierarchical and complex designs, identification of the appropriate level for tests and full reporting of outcomes
☒	☐	Estimates of effect sizes (e.g. Cohen's d, Pearson's r), indicating how they were calculated

Our web collection on statistics for biologists contains articles on many of the points above.

Software and code

Policy information about availability of computer code

Data collection	No software was used for data collection.

Data analysis	Software used in this study is generally open source, and all publicly available. Full descriptions, including parameters and version numbers, are provided in the Materials & Methods, and further detail on the bioinformatics protocols can be found at: https://github.com/Psy-Fer/SARS-CoV-2_GTG.<br><br>Version numbering was as follows:<br>bwa mem 0.7.12-r1039<br>iVar 1.0<br>samtools mpileup v1.9<br>bcftools call v1.9<br>RAMPART 1.0.6<br>Guppy 4.0.14<br>minimap2 2.17-r941<br>Medaka 0.11.5<br>LongShot 0.4.1<br>Varscan2 v2.4.3<br>bcftools norm 1.9<br>rtg-tools vcfdecompose 3.10.1<br>gatk LeftAlignAndTrimVariants 4.0.11.0<br>NGMLR v0.2.7<br>Sniffles v1.0.11

For manuscripts utilizing custom algorithms or software that are central to the research but not yet described in published literature, software must be made available to editors and reviewers. We strongly encourage code deposition in a community repository (e.g. GitHub). See the Nature Research guidelines for submitting code & software for further information.

Data

Policy information about availability of data

All manuscripts must include a data availability statement. This statement should provide the following information, where applicable:

- Accession codes, unique identifiers, or web links for publicly available datasets
- A list of figures that have associated raw data
- A description of any restrictions on data availability

Raw data has been deposited to the Sequence Read Archive under Bioproject PRJNA675364. Consensus genomes for the majority of samples sequenced here have been deposited separately to GISAID (see Supplementary Data 1). Source data are provided with this paper as Source Data file.

Field-specific reporting

Please select the one below that is the best fit for your research. If you are not sure, read the appropriate sections before making your selection.

[x] Life sciences [ ] Behavioural & social sciences [ ] Ecological, evolutionary & environmental sciences

For a reference copy of the document with all sections, see nature.com/documents/nr-reporting-summary-flat.pdf

Life sciences study design

All studies must disclose on these points even when the disclosure is negative.

Sample size	As a benchmarking / proficiency testing study, large sample sizes and high replication was essential. All sample sizes are clarified in the text, both in terms of the number of unique samples analysed and the number of variants analysed, for example:<br>"In total, we obtained complete (99.6%) genome coverage with both technologies for 157 matched positive cases (Supplementary Table 1)... this provides an overall comparison set of 1201 consensus variants and 4,674,554 positions that match the reference strain in a given sample"
Data exclusions	Specimens that could not be completely amplified by PCR and, hence, sequenced were not included in the study.
Replication	As a benchmarking / proficiency testing study, large sample sizes and high replicate was essential. The number of replicates used in a given analysis is clarified in the main text and also within the relevant figure legends, for example:<br>"Eight independent replicates were sequenced on ONT PromethION and Illumina MiSeq instruments (see Methods)."
Randomization	Specimens selected for inclusion in the study were chosen at random, with age, sex, disease severity etc not available to researchers. The only selection criteria was the requirement for SARS-CoV-2 viral titre of Ct = 30 or less.
Blinding	All data analysis is performed using standardized bioinformatics workflows, the outcomes of which cannot be influenced by the biases of participants or researchers. Therefore, blinding was not relevant to the study.

Reporting for specific materials, systems and methods

We require information from authors about some types of materials, experimental systems and methods used in many studies. Here, indicate whether each material, system or method listed is relevant to your study. If you are not sure if a list item applies to your research, read the appropriate section before selecting a response.

Materials & experimental systems

n/a	Involved in the study
☒	☐ Antibodies
☒	☐ Eukaryotic cell lines
☒	☐ Palaeontology and archaeology
☒	☐ Animals and other organisms
☐	☒ Human research participants
☒	☐ Clinical data
☒	☐ Dual use research of concern

Methods

n/a	Involved in the study
☒	☐ ChIP-seq
☒	☐ Flow cytometry
☒	☐ MRI-based neuroimaging

Human research participants

Policy information about studies involving human research participants

Population characteristics	De-identified patient specimens from individuals testing positive for SARS-CoV-2 in New South Wales, Australia, during March-April 2020 were used in this study. Specimens selected for inclusion in the study were chosen at random, with age, sex, disease severity etc not available to researchers. The only selection criteria was the requirement for SARS-CoV-2 viral titre of Ct = 30 or less.
Recruitment	Specimens were collected from patients testing positive to SARS-CoV-2 by NSW Health Pathology East Serology and Virology Division (SaViD). All specimens were nasopharyngeal swabs.
Ethics oversight	HREC at South Eastern Sydney Local Health District (SESLHD) approval is 2020/ETH00287

Note that full information on the approval of the study protocol must also be provided in the manuscript.
